# USP1 Regulates TAZ Protein Stability Through Ubiquitin Modifications in Breast Cancer

**DOI:** 10.3390/cancers12113090

**Published:** 2020-10-23

**Authors:** Ashley Mussell, He Shen, Yanmin Chen, Michalis Mastri, Kevin H. Eng, Wiam Bshara, Costa Frangou, Jianmin Zhang

**Affiliations:** 1 Department of Cancer Genetics & Genomics, Roswell Park Comprehensive Cancer Center, Elm and Carlton Streets, Buffalo, NY 14203, USA; ashley.mussell@roswellpark.org (A.M.); He.shen@roswellpark.org (H.S.); yanmin.chen@roswellpark.org (Y.C.); michalis.mastri@roswellpark.org (M.M.); kevin.eng@roswellpark.org (K.H.E.); 2 Department of Pathology, Roswell Park Comprehensive Cancer Center, Elm and Carlton Streets, Buffalo, NY 14263, USA; wiam.bshara@roswellpark.org; 3 Harvard T.H. Chan School of Public Health, Molecular and Integrative Physiology Department, 665 Huntington Ave., Boston, MA 02115, USA; cfrangou@hsph.harvard.edu

**Keywords:** USP1, TAZ, triple negative breast cancer

## Abstract

**Simple Summary:**

Triple-Negative breast cancer (TNBC) is the most aggressive form of breast cancer in women. Targeted therapies for the treatment of this disease are severely lacking. Through mechanistic studies of the key component of Hippo signaling pathway, Transcriptional co-activator with PDZ-binding motif (TAZ), we aimed to uncover novel regulators that may be used as targeted therapies for this disease. Using an siRNA target deubiquitinating enzymes screen, we identified ubiquitin-specific peptidase 1 (USP1) as a novel TAZ deubiquitinating enzyme. We found that USP1 interacts with TAZ and loss of USP1 reduces cell proliferation in a partially TAZ-dependent manner. Furthermore, we demonstrated that USP1 and TAZ expression are positively correlated in TNBC patients. This research found a newly defined regulatory mechanism of TAZ that could be used as a therapeutic approach for breast cancer.

**Abstract:**

The Hippo signaling pathway is an evolutionarily conserved pathway that was initially discovered in *Drosophila melanogaster* and was later found to have mammalian orthologues. The key effector proteins in this pathway, YAP/TAZ, are often dysregulated in cancer, leading to a high degree of cell proliferation, migration, metastasis and cancer stem cell populations. Due to these malignant phenotypes it is important to understand the regulation of YAP/TAZ at the protein level. Using an siRNA library screen of deubiquitinating enzymes (DUBs), we identified ubiquitin specific peptidase 1 (USP1) as a novel TAZ (WWTR1) regulator. We demonstrated that USP1 interacts with TAZ and increases TAZ protein stability. Conversely, loss of function of USP1 reduces TAZ protein levels through increased poly-ubiquitination, causing a decrease in cell proliferation and migration of breast cancer cells. Moreover, we showed a strong positive correlation between USP1 and TAZ in breast cancer patients. Our findings facilitate the attainment of better understanding of the crosstalk between these pathways and may lead to potential therapeutic interventions for breast cancer patients.

## 1. Introduction

Breast cancer (BC) is the leading cause of cancer incidence and the second leading cause of cancer-related mortality in women in the United States [1,2]. BC is a heterogeneous disease that can be classified into at least four major molecular subtypes based on gene expression profiles: luminal A, luminal B, HER2-enriched and basal-like triple negative breast cancer (TNBC) [3]. Basal-like breast cancers are associated with aggressive pathological features and poor clinical outcomes [4].

Despite the extensive advancements in targeted therapies for breast cancer, the effective treatment of basal-like breast cancers remains a major challenge [5]. The current standard of care for basal-like breast cancers is restricted to conventional chemotherapy, as they do not respond to endocrine treatments or targeted therapies [6]. To achieve better treatment of these neoplasia’s new targets must be identified.

The Hippo signaling effector proteins YAP/TAZ are associated with multiple cancer types and are specifically amplified or upregulated in TNBC [7,8,9,10]. Due to the fact that there are no germline mutations present in the YAP/TAZ chromosomal regions, the mechanism through which these proteins are increased in TNBCs needs to be better understood [11,12]. TAZ plays a critical role in cell migration, invasion, tumorigenesis and stem cell traits in breast cancer cells [13,14]. TAZ is not only a driver of basal-like breast cancer progression and a novel prognostic factor but is also required for the maintenance of tumor growth and established metastases [15].

Previous studies have focused on individual components of the Hippo signaling pathway but the means by which external pathways alter Hippo pathway proteins is under-studied. It is known that aberrations in upstream regulators affect YAP/TAZ activity and expression levels. For instance, the phosphorylation of TAZ by upstream kinase LATS leads to the subsequent phosphorylation by CK1Ɛ and the recruitment of SCF^β-TrCP^ E3 ligase to promote TAZ degradation through the 26S proteasome [16]. Since there are no known drugs that can target TAZ at the protein level, the therapeutic intervention points in TAZ-driven malignancies outside of canonical Hippo signaling should be determined.

Post-translational modifications are enzymatic processes that modify proteins after biosynthesis [17]. The addition of ubiquitin is one of the most common post-translational modifications, marks proteins for degradation, alters their functions or changes their signaling processes [18]. Through a multi-step process, ubiquitin moieties are added to the proteins, thereby changing their fate. As with most cellular processes, ubiquitination is reversible through the action of deubiquitinating enzymes (DUBs). DUBs are a family of enzymes that remove the ubiquitin from target substrates to increase stability, change cellular localization or modify protein-protein interactions [19,20,21]. Multiple reviews have focused on how E3 ligases act on the Hippo signaling pathway but the deubiquitinating enzymes that affect Hippo components, specifically YAP/TAZ, are largely unknown [22,23]. Recently, USP10 was found to stabilize YAP/TAZ protein levels in hepatocellular carcinoma; however, there are currently no known DUBs that regulate TAZ specifically in breast cancer [24].

To assess the novel regulators of TAZ at the protein level, we used an siRNA library screen of DUBs and identified that USP1 can alter TAZ stability in breast cancer cells. We found that the loss of USP1 reduces TAZ protein levels through increased TAZ poly-ubiquitination. Genetic perturbation of USP1 alters cell proliferation and migration in a TAZ-dependent manner. Furthermore, we demonstrated a link between USP1 and TAZ protein levels in TNBC. These findings uncovered a novel regulatory mechanism of TAZ that could serve as a biomarker for the treatment or prognosis in BC patients.

## 2. Results

### 2.1. USP1 Is a Novel DUB of TAZ

**Figure 1:**
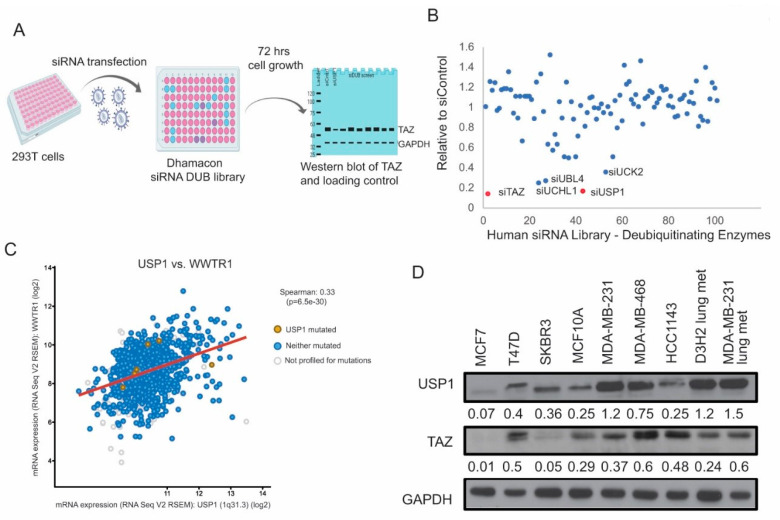
USP1 is a novel DUB of TAZ. (**A**) Schematic representation of siRNA screen using Dharmacon siRNA DUB library prepared using BioRender. Three siRNAs targeting a single DUB were transfected per well. (**B**) Dot plot quantification of relative TAZ expression upon transfection with siRNA library from immunoblots. (**C**) Spearman correlation plot of mRNA expression of WWTR1 vs. USP1 from TCGA data. (**D**) Immunoblot analysis of USP1 and TAZ expression in a panel of breast cancer cell lines (MCF7 and T47D – luminal, SKBR3 – HER2 overexpression, MCF10A – non-transformed epithelial, MDA-MB-231, MDA-MB-468 and HCC1143, D3H2 lung metastasis of MDA-MB-231 cells– TNBC). GAPDH was used as a loading control. Immunoblots were quantified and normalized to GAPDH.

We employed an siRNA screen targeting genome-wide DUB enzymes to understand how deubiquitinating enzymes alter TAZ protein levels in vitro. The TAZ-expressing construct was co-transfected with the Dharmacon siRNA DUB library in HEK293T cells. The siControl or siTAZ served as negative or positive control. Protein lysates were collected 72 h post-transfection and exogenous TAZ expression was detected by immunoblot (Figure 1A). We found that the knockdown of several DUBs such as, USP1, UCK2, UBL4A and UCHL1 dramatically reduced TAZ protein levels (Figure 1B). Using TCGA data, we found that USP1 and TAZ significantly co-occurred in the TNBC cases, while the other DUBs had a less significant co-occurrence (USP1:WWTR1 OR = 2.958, *p* < 0.001; UCK2:WWTR1 OR = 1.605, *p* < 0.001; UBL4A:WWTR1 OR = 1.123, *p* < 0.013; UCHL1:WWTR1 OR = 1.497, *p* < 0.009) [25,26]. Using mRNA expression data, we also found that USP1 and TAZ were positively correlated (Figure 1C, R = 0.33, *p* < 0.001). The other DUBs identified in our screen had a much lower Spearman correlation than that of USP1 and TAZ (UCK2: rho = 0.18; UBL4A: rho = −0.13; UCHL1: rho = 0.29). Since USP1 is known to act in an oncogenic manner while UCHL1 and UBL4A are shown to have tumor-suppressive functions, we focused our studies on how USP1 alters TAZ [27,28,29]. To understand the expression of USP1 and TAZ at the protein level, we analyzed a panel of breast cancer cell lines. Interestingly, we found that USP1 and TAZ were highly expressed in TNBC cell lines (MDA-MB-231, MDA-MB-468) when compared to non-transformed (MCF10A) or luminal cell lines (MCF7, T47D) (Figure 1D). Altogether, these data showed that USP1 and TAZ protein levels are positively correlated in TNBC.

### 2.2. USP1 Acts as a Post-Translational Modifier of TAZ

**Figure 2:**
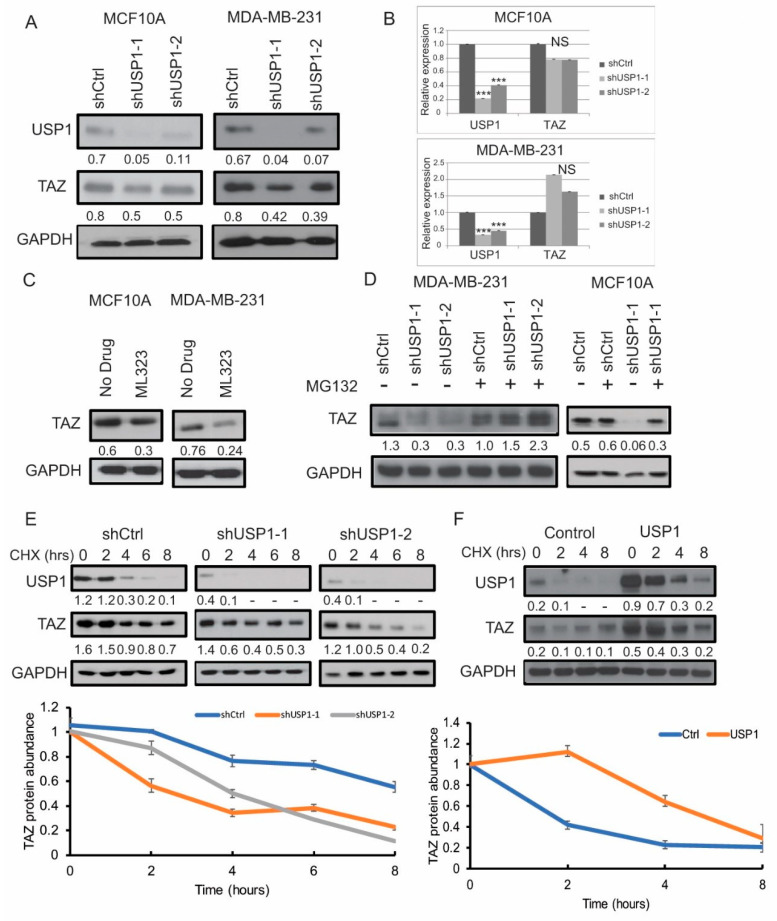
USP1 acts as a post-translational modifier of TAZ. (**A**) Immunoblot analysis for USP1 and TAZ expression in USP1 knockdown MCF10A and MDA-MB-231 cells. GAPDH was used as a loading control. (**B**) Relative mRNA expression of USP1 and TAZ in USP1 knockdown MCF10A and MDA-MB-231 cells. (**C**) Immunoblot analysis for TAZ expression in MCF10A and MDA-MB-231 upon treatment with 15µM ML323. GAPDH was used as a loading control. (**D**) Immunoblot analysis for TAZ expression in USP1 knockdown MCF10A and MDA-MB-231 cells with MG132treatment. GAPDH was used as a loading control. (*n* = 2) (**E**) Immunoblot analysis for USP1 and TAZ in USP1 knockdown MDA-MD-231 cells with CHX treatment at various time points. GAPDH was used as a loading control. (*n* = 3) (**F**) Immunoblot analysis for USP1 and TAZ expression in USP1 overexpressing MCF7 cells with CHX treatment at various time points. GAPDH was used as a loading control. (*n* = 2) Data are shown as the mean ± SD. Unpaired two-tailed student t-test: * *p* < 0.05, ** *p* < 0.01, *** *p* < 0.001. NS: no significance. Immunoblots were quantified and normalized to GAPDH.

Next, to confirm our initial findings, we assessed whether the loss of USP1 affects TAZ protein levels. The knockdown of USP1 in MCF10A and MDA-MB-231 cells using two independent shRNAs against USP1 showed a reduction in TAZ protein levels, with no change in mRNA levels, indicating that the reduction of TAZ protein level was not from alterations at the transcription level (Figure 2 A,B). We also noted no change in other Hippo components upon knockdown of USP1 in MDA-MB-231 cells (Figure S1A). USP1 activity is dependent on its interaction with UAF1 [30]. To validate our USP1 knockdown result, we used a small molecule that targets the USP1/UAF complex. We first determined the IC_50_ of ML323, a USP1/UAF1 complex inhibitor, on various cell lines (Figure S1B). Consistent with the genetic knockdown of USP1, we found a reduction of TAZ protein levels in response to ML323 treatment (Figure 2C).

Upon ubiquitination, proteins are targeted to the proteasome for degradation. To determine whether the reduction of the TAZ protein levels in shUSP1 occurs through proteasome degradation, we treated the shControl or shUSP1 cells with the proteasome inhibitor MG132. Accordingly, we found that the proteasome inhibitor treatment significantly rescued the TAZ protein levels in the absence of USP1, indicating that USP1 stabilizes TAZ and protects it from proteasome degradation (Figure 2D).

To determine whether the loss of USP1 function affects TAZ protein half-life, we treated the shControl or shUSP1 MDA-MB-231 cells with cycloheximide, a drug that is widely used for inhibiting protein synthesis [31]. We found that the TAZ protein half-life is dramatically reduced in the shUSP1 cells compared to the shControl cells (about 4 h in the absence of USP1 compared to 8 h when USP1 is present) (Figure 2E). We transduced MCF7 cells with a USP1 overexpression construct to assess how USP1 overexpression affects TAZ stability. Consistently, overexpressing USP1 significantly increased TAZ protein stability (about 6 h under USP1 overexpression condition compared to 2 h under the control condition) (Figure 2F). Taken together, these results demonstrate that USP1 expression increases TAZ protein half-life.

### 2.3. USP1 and TAZ form a Complex That Alters TAZ Ubiquitination

**Figure 3:**
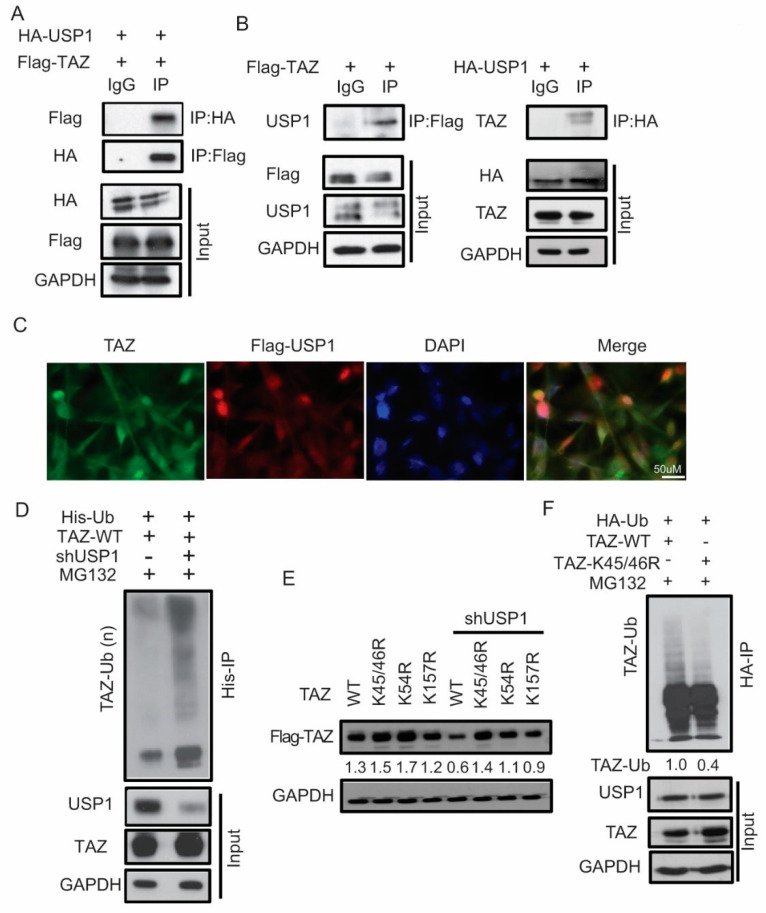
USP1 and TAZ form a complex and alters TAZ ubiquitination. (**A**) Interaction between exogenous USP1 and TAZ in 293T cells. Cellular extracts were immunoprecipitated with Flag or HA beads and probed for antibodies against indicated proteins. GAPDH was used as a loading control. (**B**) Interaction between exogenous TAZ and endogenous USP1 or exogenous USP1 and endogenous TAZ. GAPDH was used as a loading control. (**C**) Immunofluorescence staining of Flag-USP1 and TAZ in MDA-MB-231 cells (Scale bar = 50 µm). (**D**) Increased ubiquitination of TAZ upon knockdown of USP1 and immunoprecipitation with His-beads. GAPDH was used as a loading control for input. (**E**) Immunoblot analysis of TAZ-WT or lysine mutants in the presence or absence of USP1. GAPDH was used as a loading control. (**F**) Decreased ubiquitination of K45/46R mutation after co-immunoprecipitation with HA-beads. GAPDH was used as a loading control for input. Immunoblots were quantified and normalized to GAPDH.

To determine whether USP1 interacts with TAZ, we co-transfected Flag-TAZ and HA-USP1 into HEK293T cells and co-immunoprecipitation (co-IP) assays were performed. Immunoprecipitation with Flag antibody showed that Flag-TAZ can interact with HA-USP1 proteins (Figure 3A). Alternatively, immunoprecipitation with HA antibody showed that HA-USP1 reciprocally interacts with Flag-TAZ (Figure 3A). Furthermore, we confirmed that both exogenous TAZ or USP1 can interact with endogenous USP1 or TAZ (Figure 3B). In addition, we detected that USP1 and TAZ colocalize in MDA-MB-231 cells by immunofluorescence detection. Using anti-Flag antibody (red) and anti-TAZ antibody (green), we show that USP1 (exogenous) and TAZ (endogenous) tend to co-localize in the nucleus at high density conditions, which is typical of MDA-MB-231 cells (Figure 3C).

Next, to determine whether the loss of USP1 affected TAZ ubiquitination, we co-transfected His-tagged ubiquitin (His-Ub) and TAZ in the presence or absence of USP1 and treated with proteasome inhibitor MG132. The His-IP assays confirmed that TAZ ubiquitination is increased in the absence of USP1 (Figure 3D). Protein ubiquitination begins with the attachment of a single ubiquitin molecule to a substrate lysine residue. Evolutional evaluation of TAZ showed that lysine 45/46 is highly conserved across species, whereas other lysine residues were not (Figure S2). To determine the lysine residue in TAZ that may be ubiquitinated, we transfected several TAZ lysine mutants in the presence or absence of USP1. We found TAZ-WT, -K54R and -K157R mutants showed a slight reduction in TAZ protein levels in the absence of USP1, whereas TAZ-K45/46R mutant protein levels were not altered (Figure 3E). The result suggested that ubiquitination of TAZ occurs predominantly on K45/46. We then co-transfected HA-Ub and TAZ-WT or -K45/46R TAZ in HEK293T cells, HA-Ub immunoprecipitation assay demonstrated the decreased ubiquitination of TAZ in the TAZ-K45/46R mutant compared to TAZ-WT (Figure 3F). Taken together, these results suggest that USP1 directly interacts with and deubiquitinates TAZ and that ubiquitination predominantly occurs at TAZ lysine 45/46.

### 2.4. USP1 Depletion Impairs Breast Cell Proliferation in a Partially TAZ Dependent Manner

**Figure 4:**
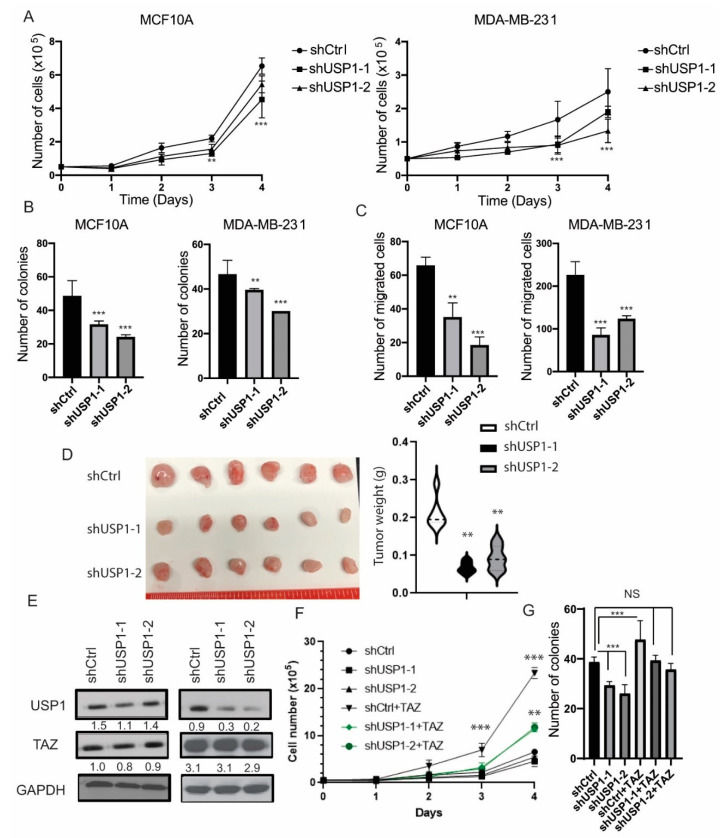
Loss of function of USP1 alters cell proliferation and migration. (**A**) Cell proliferation assay of MCF10A and MDA-MB-231 cells upon knockdown of USP1. (**B**) Quantification of clonogenicity assays after 11 days upon knockdown of USP1 in MCF10A and MDA-MB-231 cells. (**C**) Quantification of cell migration after knockdown of USP1 in MCF10A and MDA-MB-231 cells. (**D**) Images and quantification of tumor size of shCtrl, shUSP1-1 or shUSP1-2 MDA-MB-231 cells injected into the mammary fat pad of SCID mice, *n* = 6. (**E**) Immunoblot analysis of TAZ expression in shUSP1 MCF10A cells. GAPDH was used as a loading control. (**F**) Cell proliferation of control or TAZ-4SA transduced shUSP1 MCF10A cells. (**G**) Quantification of clonogenicity assay after 11 days of control or TAZ-4SA transduced shUSP1 MCF10A cells. Data are shown as the mean ± SD. Unpaired two-tailed student t-test: * *p* < 0.05, ** *p* < 0.01, *** *p* < 0.001. Western blots were quantified and normalized to GAPDH.

Many studies have shown that TAZ promotes the growth of non-transformed and transformed mammary epithelial cells [32,33]. Therefore, to assess how the loss of function of USP1 affects cell proliferation, we knocked-down USP1 using two independent shRNAs targeting USP1 in MCF10A and MDA-MB-231 cells. USP1 depletion reduced cell proliferation in both MCF10A and MDA-MB-231 cell lines (Figure 4A). Furthermore, the knockdown of USP1 corresponded to a reduction in clonogenicity and cell migration (Figure 4B,C). To determine the effects of knockdown USP1 in vivo, we injected shCtrl or shUSP1 MDA-MB-231 cells into the mammary fat pad of SCID mice. We found that USP1 knockdown cells had a significant reduction in tumor growth compared to control cells, indicating that USP1 loss reduces cell growth both in vitro and in vivo (Figure 4D).

To determine whether shUSP1 reduced cell proliferation through the effects of TAZ, we performed rescue experiments. We transduced the shUSP1 MCF10A cells with exogenous TAZ-4SA (constitutively active TAZ) (Figure 4E). The re-expression of TAZ induced cell proliferation and clonogenicity phenotypes (Figure 4F,G). Taken together, these results suggest that loss of function of USP1 reduced cell proliferation, in part, through dysregulation of TAZ.

**Figure 5:**
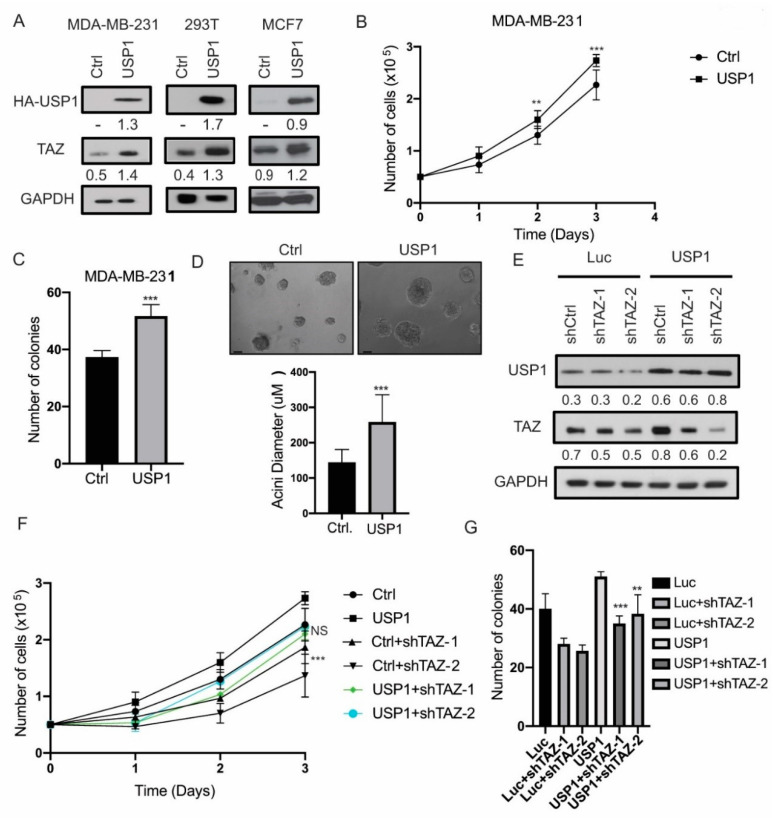
USP1 overexpression increases cell proliferation in a partially TAZ-dependent manner. (**A**) Immunoblot analysis for USP1 and TAZ in USP1 transduced MDA-MB-231, 293T and MCF7 cells. GAPDH was used as a loading control. (**B**) Cell proliferation assay of USP1 overexpressing MDA-MB-231 cells. (**C**) Quantification of clonogenicity assay after 11 days in USP1 overexpressing MDA-MB-231 cells. (**D**) Representative images and quantification of spheroid size in control or USP1 overexpressing MDA-MB-231 cells (Scale bar = 50 µm). (**E**) Immunoblot analysis of TAZ knockdown in USP1 overexpressing MDA-MB-231 cells. GAPDH was used as a loading control. (**F**) Cell proliferation assay for shCtrl or shTAZ in USP1 overexpressing MDA-MB-231. (**G**) Quantification of clonogenicity assay after 11 days for shCtrl or shTAZ in USP1 overexpressing MDA-MB-231. Data are shown as the mean ± SD. Unpaired two-tailed student t-test: * *p* < 0.05, ** *p* < 0.01, *** *p* < 0.001. Immunoblots were quantified and normalized to GAPDH.

Next, to evaluate the effects of USP1 overexpression on TAZ, we transduced HEK293T, MDA-MB-231 and MCF7 cells with USP1. The overexpression of USP1 led to a significant increase of TAZ protein levels in all cell lines (Figure 5A). Furthermore, USP1 overexpression increased cell proliferation and clonogenicity in MDA-MB-231 cells (Figure 5B,C). In addition, we assessed the effects of USP1 overexpression in 3D culture. While we did not observe an increase in the overall number of spheroids formed, we found a significant increase in spheroid size (Figure 5D). Finally, to test whether TAZ is essential for USP1 proliferative phenotypes, we transduced USP1 overexpressing MDA-MB-231 with two independent hairpins targeting TAZ (Figure 5E). Consistent with our previous finding, we showed that the knockdown of TAZ reduced cell proliferation and clonogenicity of USP1 overexpressing cells (Figure 5F,G). Taken together these results showed that USP1 promotes cell proliferation in a partially TAZ-dependent manner.

### 2.5. USP1 and TAZ Expression Are Correlated in TNBC Patients

**Figure 6:**
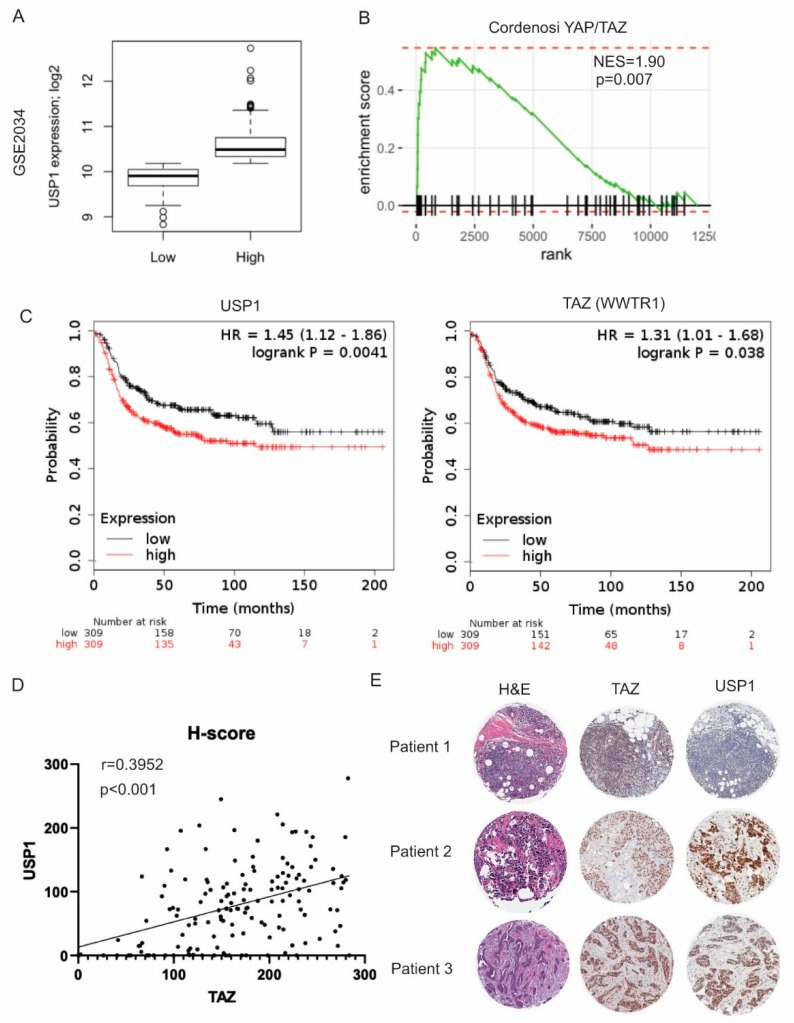
USP1 and TAZ expression are correlated in TNBC. (**A**) Stratification of GSE2034 into USP1 low- and USP1 high- subsets. (**B**) Cordenosi YAP/TAZ gene signature is highly correlated with USP1 high-subset patients. (**C**) Kaplan-Meier relapse free survival analysis curves for USP1 and TAZ in breast cancer patients. (**D**) The dot plot represents the correlation of USP1 and TAZ in all the TMAs stained indicating a positive correlation between staining intensity of ALL samples. Pearson correlation between USP1 and TAZ protein levels were quantified by Aperio Image Scope of TNBC patients treated at RPCCC. Data are shown as the mean ± SD. Unpaired two-tailed student t-test: * *p* < 0.05, ** *p* < 0.01, *** *p* < 0.001. (**E**) Representative images of H&E and IHC staining of tumor microarrays (TMAs) from TNBC patients treated at Roswell Park Comprehensive Cancer Center.

To address whether USP1 and TAZ could be used as markers for the prognosis of breast cancer patients, we used gene set enrichment analysis (GSEA) and immunohistochemistry (IHC) staining of TNBC cases. We first stratified TNBC patients from several publicly available datasets into USP1 low- or high-expression subsets using methods previously described by Ma et al. [34]. (Figure 6A and Figure S3). Interestingly, we found that the YAP/TAZ gene signatures were significantly enriched in the USP1 high samples compared to the other gene signatures, through GSEA (Figure 6B and Figure S3) In addition, using publicly available datasets, we found that the high expression of both USP1 or TAZ is associated with a reduction in relapse-free survival in breast cancer cases (Figure 6C) [35].

We then performed TAZ and USP1 immunohistochemistry (IHC) staining in tissue microarrays (TMAs) that contained TNBC cases from Roswell Park Comprehensive Cancer Center (RPCCC) (Figure S4A). To understand the correlation of USP1 and TAZ expression in all 152 tumors stained, we plotted USP1 expression versus TAZ expression. The USP1 and TAZ protein levels were significantly positively correlated in these patient samples (Figure 6D, USP1 and TAZ, *r* = 0.3952, *p* < 0.001). Representative images of high USP1/TAZ expression (patients 2 and 3) and low USP1/TAZ expression (patient 1) tumors are shown in Figure 6E. Collectively, these data suggest that USP1 expression is correlated with TAZ expression in TNBC patients’ samples and high USP1/TAZ expression is associated with reduced overall survival of breast cancer patients.

## 3. Discussion

Understanding the external signaling nodes that converge upon and regulate TAZ can lead to better treatment options in breast cancer patients. It is known that USP1 and TAZ both have oncogenic functions in breast cancer [36,37]. In the present study, we showed that the loss of USP1 can alter the TAZ protein levels in both non-transformed and transformed mammary epithelial cells. More recently, USP1 has been found to deubiquitinate and stabilize KPNA2, leading to pro-metastatic functions in breast cancer [34]. Since both genes are often upregulated in TNBC, this signaling axis could be blocked to prevent tumor formation and metastasis. KPNA2 has been shown to cause metabolic reprograming in glioblastoma through the induction of cMYC [38]. The YAP-TAZ-TEAD axis, in coordination with cMYC, can promote the transcription of genes associated with proliferation and migration in mammary epithelial cells [39]. It would be interesting to investigate whether there is any cross talk between KPNA2 and TAZ and whether these genes can induce a pro-metastatic phenotype in concert with USP1.

The deubiquitinating enzyme USP1 is the most well-studied DUB and it has been implicated in many cancer types [27]. The most characterized functions of USP1 are evident in the multiple steps of the DNA damage repair pathway including the Fanconi anemia pathway and in the process of translesion synthesis [40,41,42]. It has been shown that USP1 regulates the mono-ubiquitination of the Fanconi anemia protein FANCD2 and deubiquitinates the proliferating cell nuclear antigen [43]. The knockout of Usp1 in mice results in genomic instability and a Fanconi anemia phenotype [44]. USP1 deubiquitinates and stabilizes ID1, ID2 and ID3, resulting in the accumulation of ID proteins in osteosarcoma [45]. The knockdown of USP1 in osteosarcoma cells reduces the expression of mesenchymal stem cell markers and initiates an osteogenic development program.

In this study we also found that mutating lysine 45 and 46 to arginine creates a TAZ protein that cannot be ubiquitinated. This mutation is in the N-terminal region of TAZ, which is highly disorganized and functions in protein-protein interactions. Furthermore, lysine 45 is the evolutionarily conserved lysine residue present in TAZ (Figure S2). Having a better understanding of this ubiquitin mutant could help researchers understand how proteins are altered in cancer. By creating stable cell lines containing this mutant and performing biological assays we may be able to understand the initial events that occur with TAZ overexpression and find a novel regulatory phenomenon that occurs in breast cancer initiation and progression.

The functions of TAZ within the nucleus have been well established; however, how TAZ functions within the cytoplasm is still not known. Research has shown that cytoplasmic TAZ is associated with self-renewal of human embryonic stem cells, while nuclear TAZ induces differentiation [46]. While this reveals the phenotypic aspects that occur in different compartments within the cell, how TAZ is shuttled between the nucleus and cytoplasm adds another layer of complexity to its regulation. Whether TAZ is transported through passive diffusion or mediated transport has been an issue plaguing the field for years. It was recently found that the 290–345 amino acid region of TAZ is required for nuclear import [47]. Further characterization of USP1 and TAZ interaction at the amino acid level may provide further insight into TAZ cytoplasmic and nuclear shuttling.

The phosphodegron present in the N-terminal region facilitates interaction with the SCF^β-TrCP^ complex, so, the interaction of TAZ and USP1 in this location may block interaction with other proteins, resulting in the increase of the TAZ protein expression observed with our findings. Blocking interaction with these factors may leave TAZ in a state that drives concomitant nuclear accumulation. Conversely, external proteins that bind to this region may also block the newly defined nuclear localization signal, reducing TAZ nuclear shuttling. Further research is needed to determine the “perfect storm” for TAZ nuclear and cytoplasmic accumulation and in which region USP1 binds to TAZ thus leading to TAZ stabilization. The dynamic interplay between protein-protein interactions and their ability to either block other interactions or drive further downstream functions could help us understand TAZ regulation and how its dysregulation can drive breast cancer.

In summary, our research identified a regulatory mechanism that could serve as a therapeutic target/strategy for tumorigenic potential. Since USP1 has been shown to regulate other targets, such as: KPNA2, that drive breast cancer metastasis, targeting this enzyme may be beneficial in the treatment of breast cancer patients, more specifically, TNBCs.

## 4. Materials and Methods

### 4.1. Cell Line and Cell Culture

MCF10A cells have previously been described and were authenticated by short tandem repeat profiling. MDA-MB-231, HEK293T and MCF7 cells were purchased from ATCC. MCF10A cells were cultured in DMEM/F12 media (Corning, New York, NY, USA) supplemented with 5% horse serum (Invitrogen, Carlsbad, CA, USA), 1% Pen/Strep, 20 ng/mL EGF (ProSpec, East Brunswick, NJ, USA), 0.5 µg/mg hydrocortisone, 100 ng/mL cholera toxin and 10 µg/mL insulin. MDA-MB-231, HEK293T and MCF7 cells were cultured in DMEM supplemented with 10% fetal bovine serum and 1% Pen/Strep. All cells were cultured in a humidified atmosphere of 95% air and 5% CO_2_ at 37 °C.

### 4.2. Plasmids and shRNA

shUSP1 constructs were generated in the pLKO.1 vector at the AgeI/EcoRI sites. USP1 overexpression construct was a gift from Wade Harper (Addgene plasmid #22596) and cloned into the pLX304 vector. HA-Ub vector was a kind gift from the Attanasov lab (Roswell Park Comprehensive Cancer Center) and His-Ub was a gift from Dr. Wang’s lab (Roswell Park Comprehensive Cancer Center). TAZ lysine mutants were generated from the pBABE-WT-TAZ vector using a site-directed mutagenesis kit (New England Biolabs, Boston, MA, USA) and PCR. Sequences for site-directed mutagenesis are found in Table 1. shRNA hairpins targeting TAZ were obtained from Broad Institute RNAi consortium and target sequences are in Table 1. Flow cytometry was used to isolate GFP positive TAZ overexpressing cells.

Lentiviral packaging: Briefly, shRNA plasmid, Δ8.9 and VSVG were co-transfected into 293T cells with Fugen transfection reagent. Viral supernatants were collected on day 3 and 4 after transfection.

### 4.3. Plasmid Transfection

For all transfections we used the PolyJet (SignaGen, Fredrick, MD, USA) transfection reagent. HEK293T cells were plated to ensure 80% confluency next day. On day 2, 10 cm dishes were transfected with 5 ug of plasmid DNA and 15 uL of PolyJet reagent in 500 uL of DMEM with no additives. Five hours post-transfection, media was replaced with DMEM supplemented with 10% FBS and 1% PenStrep. On day 3, lysate was harvested using RIPA buffer for western blot analysis or IP buffer for co-immunoprecipitation assays. If the proteasome needed to be inhibited, MG132 was added to the cells on day 3 for 4 h and lysates was harvested.

### 4.4. Immunoblot and Co-Immunoprecipitation Analysis

For immunoblot analysis, cells were lysed in RIPA buffer (Boston Bio-Products, Ashland, MA, USA) in the presence of protease and phosphatase inhibitors (Thermo-Fisher Scientific, Waltham, MA, USA) Protein concentration was determined using the Bradford protein assay. Briefly, BSA standards at varying concentrations were made to create a standard curve. Standards were made using either RIPA buffer for western blot or IP buffer for co-IP experiments. Absorbance was read at 650 nm and protein concentrations were calculated based on the slope of the standard curve. 20–30 ug of protein was loaded, separated by SDS-PAGE (Sodium Dodecyl Sulfate-Polyacrylamide Gel Electrophoresis) and then transferred onto PVDF membranes (EMD Millipore, Danvers, MA, USA). Membranes were blocked in 5% milk in TBS-T for one hour and incubated overnight at 4 °C with primary antibodies. The next day membranes were incubated with anti-mouse or anti-rabbit secondary antibody (Bio-Rad, Hercules, CA, USA). Proteins were detected using Peirce ECL western blotting substrate.

For co-immunoprecipitation cells were lysed in IP buffer (25 mM Tris-HCl, 150 mM NaCl, 1% NP-40 and 5% glycerol, pH = 7.4) supplemented with protease and phosphatase inhibitors. 500 ug of protein lysate was incubated with either HA, Flag or His tagged beads overnight at 4 °C. Lysates were boiled at 95 °C for 10 min to denature interaction from beads and run as stated above for western blots.

All western blots were quantified using ImageJ and normalized to GAPDH expression.

### 4.5. Immunofluorescence

The cells were seeded on the wells of 24-well cell culture plates containing coverslips. 24 h later, wells were washed with PBS three times and 4% paraformaldehyde was added for fixation. After permeabilization with 0.1% Triton X-100 for 10 min, cells were blocked with PBS plus 1% BSA for 1 h and incubated with primary antibodies overnight at 4 °C. Cells were washed three times with PBS and incubated with secondary antibodies (Alexa-Fluor 488 goat anti-rabbit IgG for TAZ and Alexa-Fluor 594 goat anti-mouse IgG for Flag-USP1) for 1 h at room temperature in the dark. Finally, slips were incubated with 4′,6′-diamidino-2-phenylindole for 10 min and visualized under a fluorescent microscope.

### 4.6. Cell Proliferation and Migration

For cell proliferation experiments 5.0 × 10^4^ cells were plated in a 6-well plate. At 24, 48 and 72 h cells were counted using a hemocytometer to determine cell number. Clonogenicity assays were performed by plating 200 cells in a 6-well plate and allowing growth for 11 days. Clones were then washed with PBS, fixed with 4% paraformaldehyde and stained with crystal violet.

The transwell inserts (8 µm in pore size) were used in the migration and invasion assays. For migration assay, 1.0 × 10^5^ cells were seeded in the upper chamber and 750 µl of media was added in the lower chamber. After 24 h inserts were washed with PBS, wiped with a Q-Tip, fixed with 4% paraformaldehyde and stained with crystal violet.

### 4.7. 3D Spheroid Formation

3D cultured chambers were coated with Matrigel and 4.0 × 10^3^ cells were diluted in 5% Matrigel and transferred onto the Matrigel coated chambers. Media was replaced every 4 days until visible spheroids were formed. The assays were completed in 2-independent experiments. Spheroid diameter was quantified using ImageJ.

### 4.8. Quantitative PCR

Total RNA was isolated from cells using Trizol reagent. Real-time PCR assays were performed by Power SYBR Green PCR Master Mix on the Applied Biosystems StepOnePlus Real-Time PCR system. Primer sequences are listed in Table 1.

### 4.9. In Vivo Tumor Formation

5.0 × 10^5^ shControl or shUSP1 MDA-MB-231 cells were injected into the mammary fat pad of SCID mice. Four weeks after injection tumors were isolated and measured to determine tumor size. Six mice were used in each group. The care and use of animals were performed under the rules provided by the Declaration of Helsinki and approved by the Institutional Animal Care and Use Committee of the Roswell Park Comprehensive Cancer Center (Buffalo, NY, USA), IACUC protocol number: 1203M.

Public RNA expression data was downloaded from GEO. Using edgeR and limma R packages, GSE142767 counts data were upper-quartile normalized and GSE2034 FPKM and GSE2034 affymetrix expression data were quantile normalized [48,49,50]. Data sets were separated based on USP1 expression into high and low expression. For each data set, a rank list was created based on fold change comparing high to low USP1 expression. Gene set enrichment analysis was performed using fgsea R package (http://biorxiv.org/content/early/2016/06/20/060012) for Cordenonsi YAP conserved signature from MSigDB (Systematic name: M2871) [51,52]. Normalized enrichment scores and *p*-values were adjusted for multiple comparisons and reported.

### 4.10. Immunohistochemistry Staining

Formalin-fixed paraffin embedded tissue blocks were sectioned to 5-micron thickness and subjected to IHC studies. Quality of histomorphology of tumor samples were assessed on hematoxylin and eosin (H & E) stained sections before immunostaining. Antibodies against TAZ was purchased from Cell Signaling Technology and USP1 was purchased from Abcam. Paraffin sections were placed on charged slides and IHC staining was carried out in a Dako AutostainerPlus (Carpinteria, CA, USA) as previously described [7]. Histomorphology and immunostaining results were interpreted by a board-certified pathologist.

### 4.11. Tissue Microarrays

The TMAs were built at the Pathology Resource Network at RPCCC using the pathology paraffin archives. All patients in these TMAs had surgeries to remove the primary breast cancer lesion and metastasis, when applicable, between 1996 and 2009. The staining intensity (0, 1, 2, 3) was multiplied by the percentage of cells (0, 1, 2, 3) to generate a final staining score (H-score).

### 4.12. Patient Survival Analysis

Kaplan-Meier curves were created for Relapse Free Survival (RFS) using log-rank tests to compare USP1 or TAZ expression in breast cancer patients.

### 4.13. Statistical Analysis

All data are representative of three independent experiments unless otherwise specified. *p*-values were determined using two-tailed Student’s t-tests (*p* < 0.05 *, *p* < 0.01 **, *p* < 0.001 ***).

## 5. Conclusions

We have identified USP1 as a novel regulator of TAZ. We demonstrated that USP1 interacts with TAZ and increases TAZ protein stability. Knockdown of USP1 reduces TAZ protein levels through increased poly-ubiquitination, leading to a decrease of breast cancer cell proliferation and migration in vitro and reduced tumor formation in vivo. Furthermore, we demonstrated a strong expression correlation between USP1 and TAZ in breast cancer patients. Our findings facilitate the attainment of better understanding of the crosstalk between these pathways and lead to potential therapeutic interventions for breast cancer patients.

## Figures and Tables

**Table 1:** Key resources table.

Reagent or Resource	Source	Identifier
Antibodies		
GAPDH	Cell signaling technology	Cat# 97166
TAZ	Cell signaling technology	Cat# 4883
USP1	Cell signaling technology	Cat# 8033
Flag	Sigma Aldrich	Cat# F9291
HA	Cell signaling technology	Cat# 3724
Chemicals, peptides and Recombinant proteins		
ML323 (USP1i)	Sigma Aldrich	Cat# SML-1177-25MG
Cycloheximide	Sigma Aldrich	Cat# C7698
MG132	Sigma Aldrich	Cat# 1211877-36-9
Human siGENOME siRNA Library - Deubiquitinating Enzymes	Dharmacon	Cat# G-004705-01
Anti-Flag M2 Affinity Gel	Sigma Aldrich	Cat# A2220
Dynabead His-tag	Thermo Fisher	Cat# 10103D
Pierce Anti-HA Magnetic Beads	Thermo Fisher	Cat# 88836
**Critical Commercial Assays**		
Matrigel	Corning	Cat# 3554230
Pierce ECL western blotting substrate	Thermo Fisher	Cat# 32106
RIPA lysis and extraction buffer	Thermo Fisher	Cat# 89900
Crystal Violet Solution	Sigma Aldrich	Cat# HT90132-1L
Halt Protease and Phosphatase Inhibitor	Thermo Fisher	Cat# 78441
PolyJet In vitro DNA transfection reagent	SignaGen Laboratories	Cat# SL100688
Polybrene	Sigma Aldrich	Cat# 107689
Horse Serum	Thermo Fisher	Cat# 16050122
DMEM/F12 media	Corning CellGro	Cat# MT10090CV
DMEM	Corning CellGro	Cat# 10–090-CV
Fetal Bovine Serum	Thermo Fisher	Cat# 16030074
Epidermal Growth Factor	ProSpec	Cat# CYT-1115
Insulin	Sigma Aldrich	Cat# 9011-M
Penicillin/Streptomycin	Sigma Aldrich	Cat# P4333–100ML
PVDF membranes	EMD-Millipore	Cat# IBFP0785C
**Experimental Models: Cell Lines**		
MCF10A	Zhang lab	
MDA-MB-231	ATCC	Cat# HTB-26
MDA-MB-468	ATCC	Cat# HTB-132
HEK293T	ATCC	Cat# CRL-11268
T47D	ATCC	Cat# HBT-133
SKBR3	ATCC	Cat# HTB-30
HCC1143	ATCC	Cat# CRL-2321
MDA-MB231 (D3H2)	Dr. Jia Fang’s lab (Roswell Park)	
MDA-MB-231 (lung mets)	Zhang lab	
**Restriction Enzymes, plasmids, primers and shRNAs**		
shControl		Target sequence: CAACAAGATGAAGAGCACCAA
shUSP1–1		Target sequence: CAGAGACAAACTAGATCAA
shUSP1–2		Target sequence: GCTAGTGGTTTGGAGTTTG
plx304	Addgene	Cat# 25890
Flag-HA-USP1	Addgene	Cat# 22596
USP1 antibody for IHC	Abcam	Ca# 84772
TAZ-K39R	IDT	Forward: ATGAATCCGAGGCCTAGCTCG Reverse: GACAGAGTTGAAGAGGGC
TAZ-K45/46R	IDT	Forward: TCGTGGCGGAGGAGGATCCTGCCG Reverse: GCTAGGCTTCGGATTCATGACAG
TAZ-K54R	IDT	Forward: TCTTTCTTTAGGGAGCCTGATTC Reverse: CTCCGGCAGGATCTTCTT
TAZ-K148R	IDT	Forward: CACATAGAAAGGATCACCACATGG Reverse: ATTGAGGAAGTACCTCTG
TAZ-K157R	IDT	Forward: GACCCTAGGGCGATGAAT Reverse: TTGCCATGTGGTGATTTTTTC
TAZ-K234R	IDT	Forward: CAGCAGCAGAGGCTGCGGCTTC Reverse: CTGCTGCTGAGTGGTCAG
TAZ-K392R	IDT	Forward: GCTCTGAACAGGAGTGAGCCCTTTC Reverse: AGACTCTACATCATTGAAGAG
His-6Ub	Dr. Xiajiang Wang (Roswell Park)	NA
HA-6Ub	Dr. Boyko Attanasov (Roswell Park)	NA
EcoRI-HF	New England BioLabs	Cat# R3101S
AgeI-HF	New England BioLabs	Cat# R3552S
XhoI-HF	New England BioLabs	Cat# R0146S
KpnI-HF	New England BioLabs	Cat# R3142S
HindIII-HF	New England BioLabs	Cat# R3104S
CutSmart Buffer	New England BioLabs	Cat# B7204S
USP1 qPCR primers	IDT	Forward: GCTTTGCTGCTAGTGGTTTG Reverse: GTTGGCTTTGTGCTCCATTC
TAZ qPCR primers	IDT	Forward: AGTACCCTGAGCCAGCAGAA Reverse: GATTCTCTGAAGCCGCAGTT
GAPDH qPCR primers	IDT	Forward: GGTGAAGGTCGGAGTCAACGG Reverse: GAGGTCAATGAAGGGGTCATTG
shTAZ-1		Target sequence: CCTGCCGGAGTCTTTCTTTAA
shTAZ-2		Target sequence: GAAACTGCGGCTTCAGAGAAT

## Supplementary Materials

The following are available online at https://www.mdpi.com/2072-6694/12/11/3090/s1: Figure S1: Loss of USP1 does not affect other Hippo signaling components, Figure S2: TAZ lysine conservation, Figure S3: Stratification of GSE datasets and YAP/TAZ gene signature, Figure S4: IHC staining of 152 TNBC TMAs.
