# Supplementary material for: USP1 Regulates TAZ Protein Stability Through Ubiquitin Modifications in Breast Cancer
Source: Cancers (Basel). 2020 Oct 23;12(11):3090. doi: 10.3390/cancers12113090 (PMC7690829; doi:10.3390/cancers12113090)
Supplement: Supplementary file 1 [file cancers-12-03090-s001.pdf]

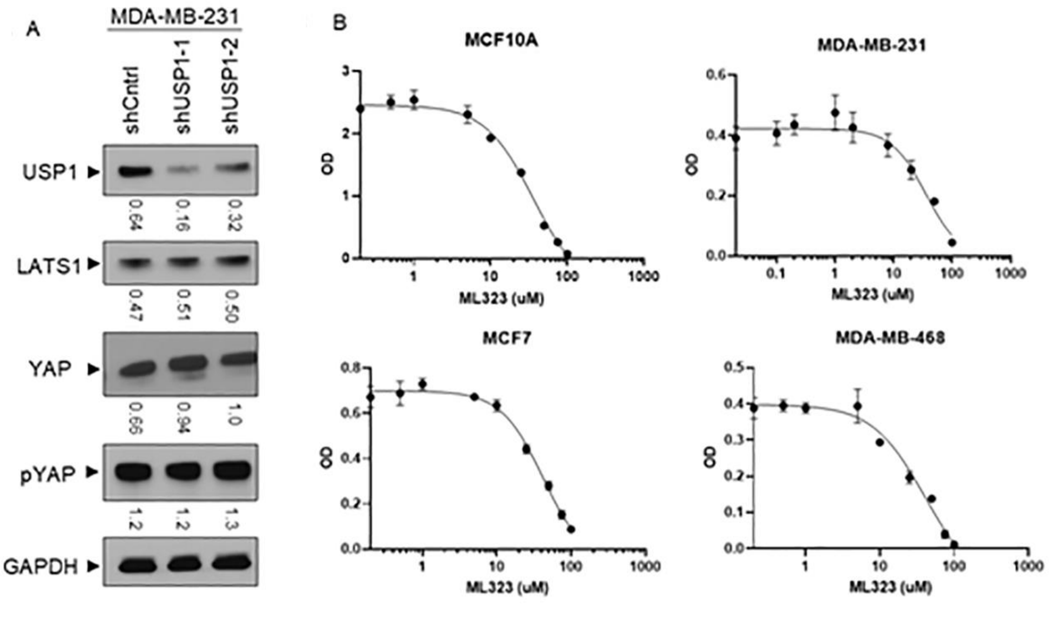

Figure S1. (**A**) Western blot analysis of MDA-MB-231 cells with USP1 knockdown and analysis for indicated antibodies. GAPDH was used as a loading control and blots were normalized to GAPDH. (**B**) Cytotoxicity experiments using 4 different cell lines and varying concentrations of ML323 (USP1/UAF1i).

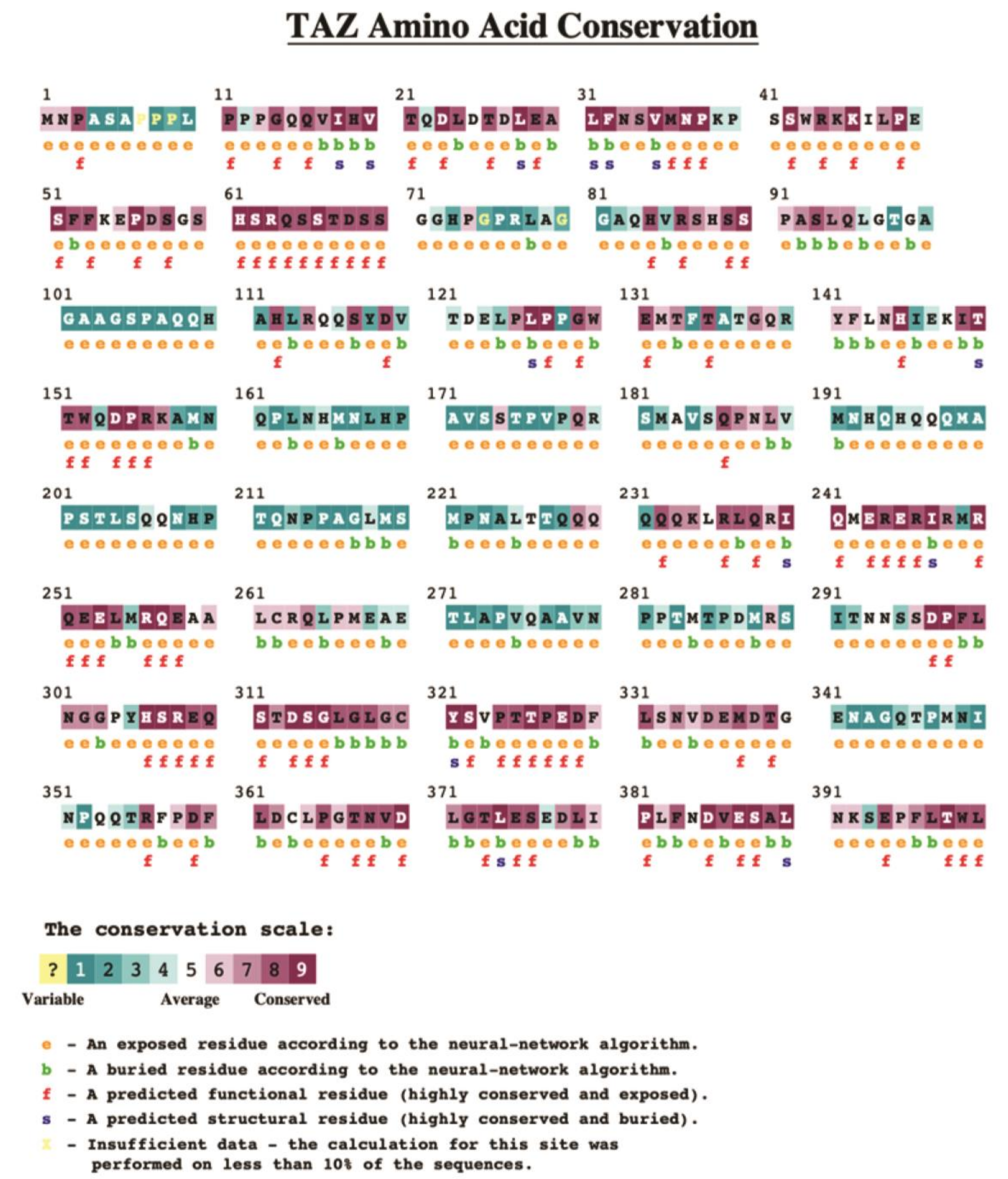

Figure S2. TAZ conservation across species.

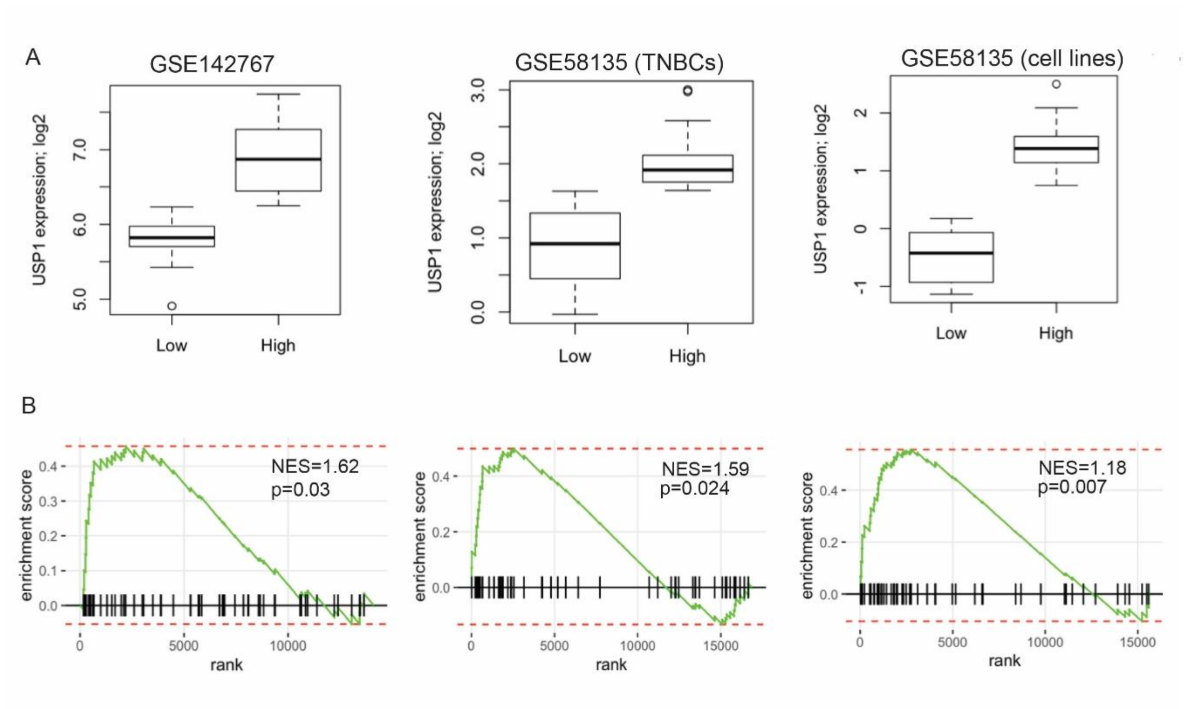

Figure S3. Stratification of GSE58135 (TNBCs), GSE58135 (Cell lines), and GSE142767 into USP1 low and USP1 high subsets. Cordenosi YAP/TAZ gene signature is shown.

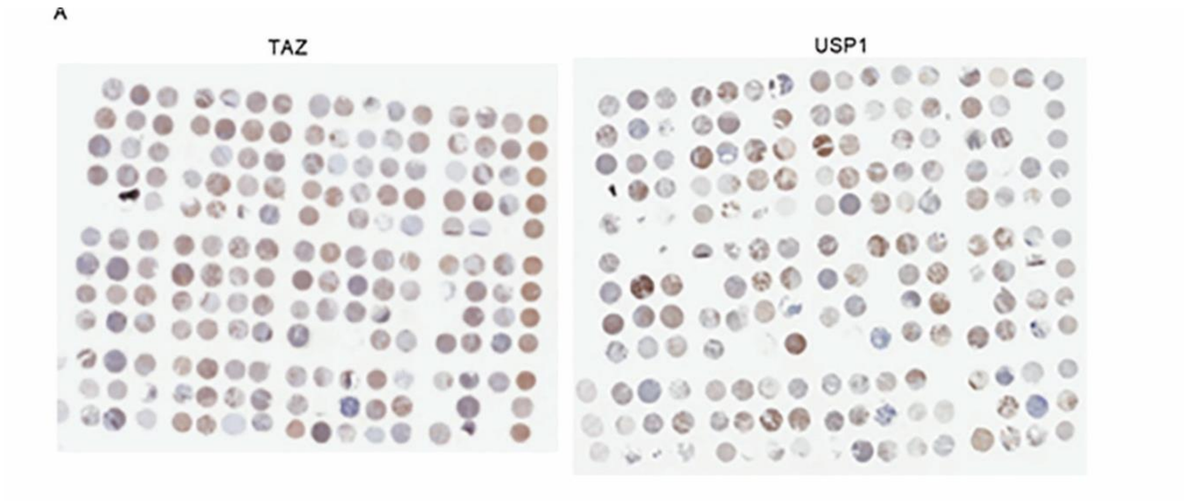

Figure S4. Representative images of TMAs stained with USP1 or TAZ antibody.

Details of Western Blot

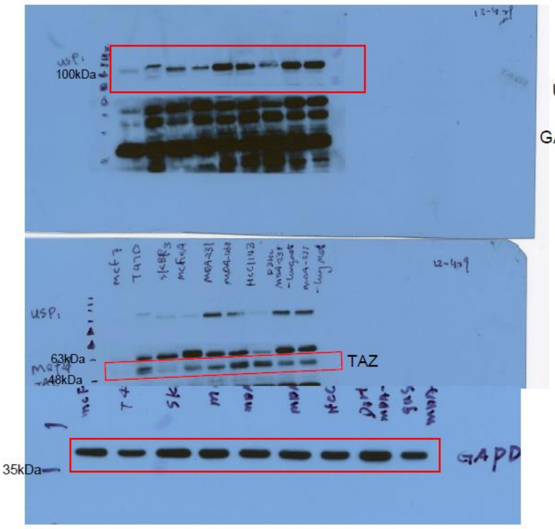

Figure 1D: Breast Cell panel

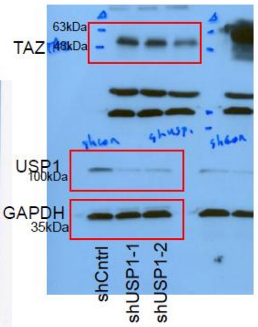

Figure 2A: shUSP1 in MCF10A

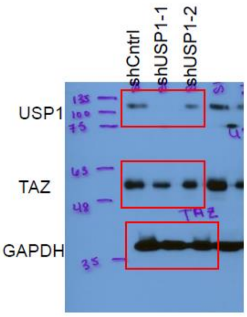

Figure 2A: shUSP1 in MDA-MB-231

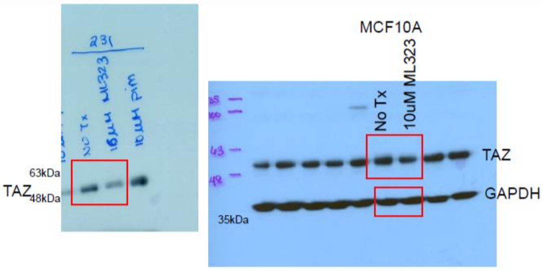

Figure 2C: ML323 treatment

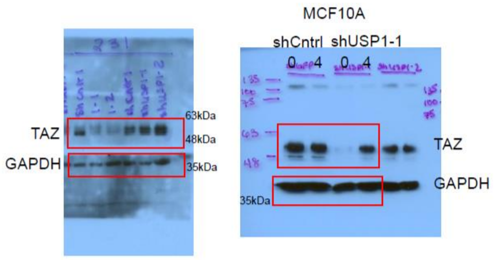

Figure 2D: MG132 TX

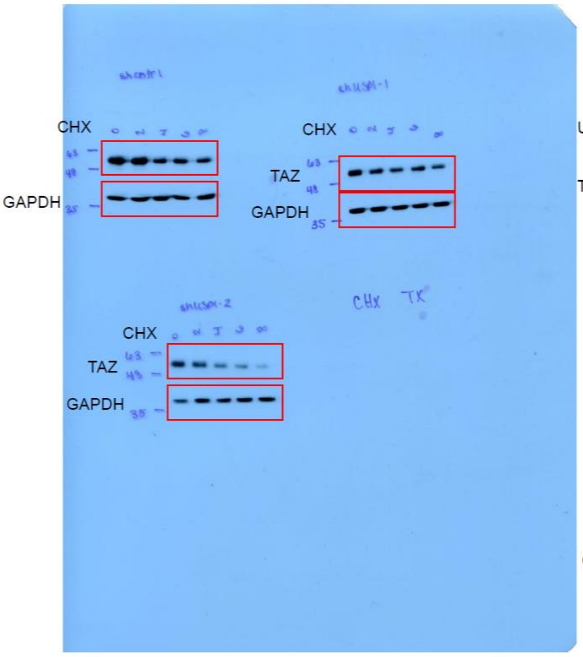

Figure 2E: CHX TX in shUSP1 MDA-MB-231

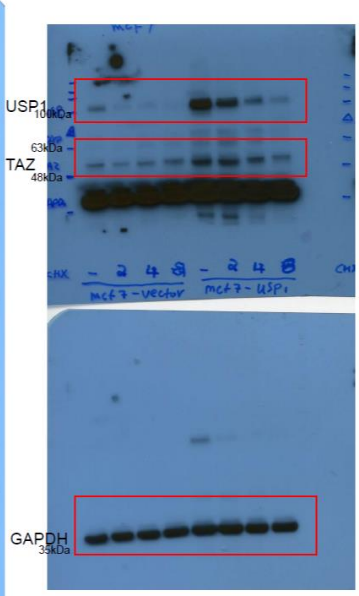

Figure 2F: CHX TX in OEUSP1 MCF7

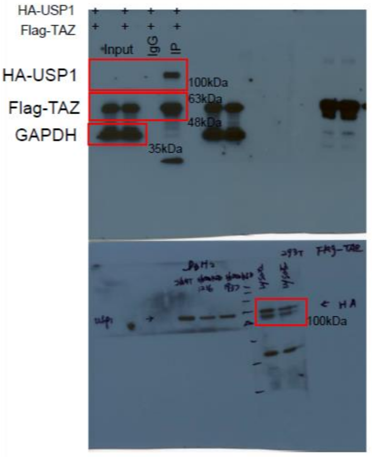

Figure 3A: Flag-TAZ_Flag-IP

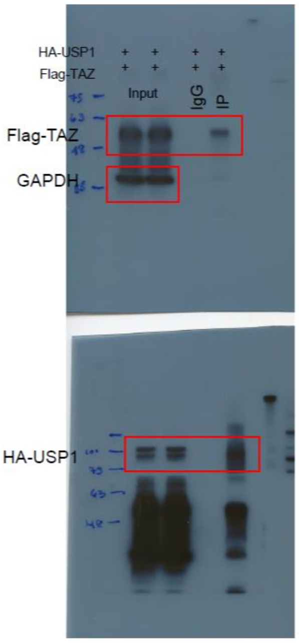

Figure 3A: HA-USP1_HA-IP

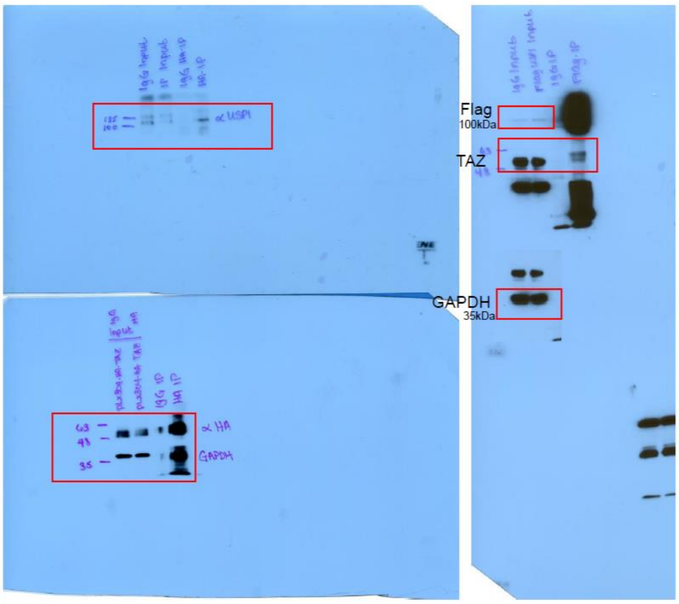

Figure 3B: Original_Exogenous
with endogenous interaction

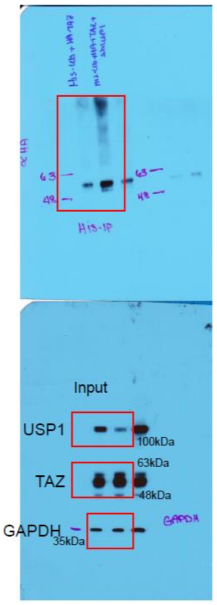

Figure 3C: Original (His-IP of
TAZ-Ub with and without USP1)

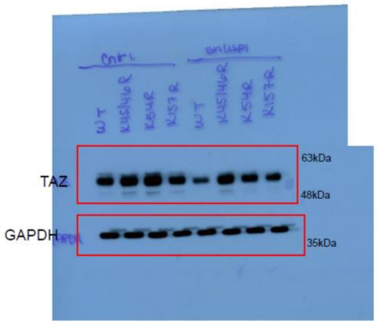

Figure 3E: Lysine mutants (No MG132)

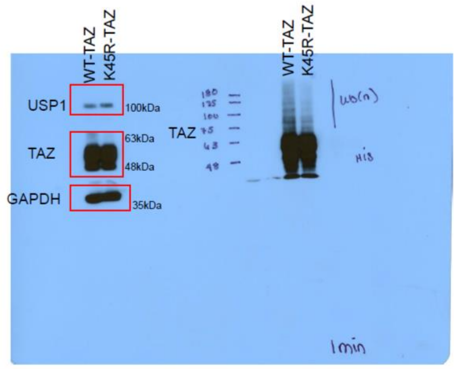

Figure 3F: Ub-IP Lysine mutant

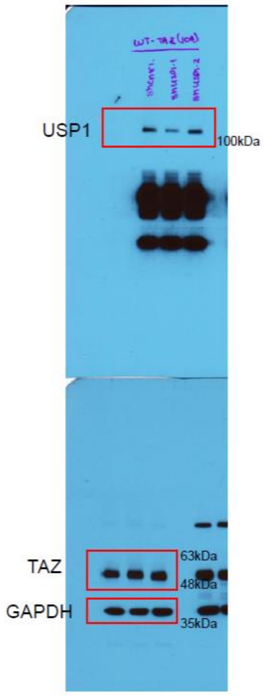

Figure 4E: WT-TAZ OE in shUSP1 MCF10A

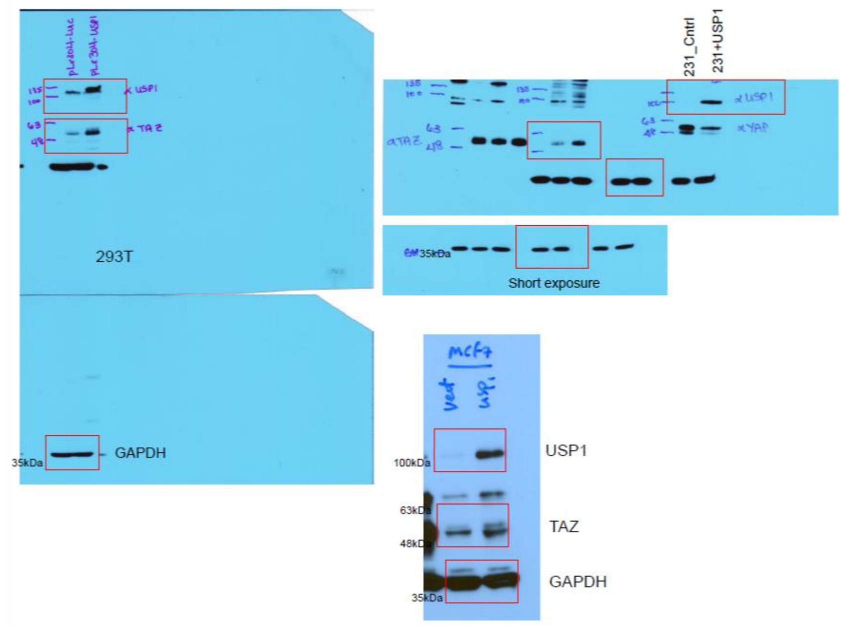

Figure 5A: USP1 OE in 293T, MDA-MB-231, and MCF7

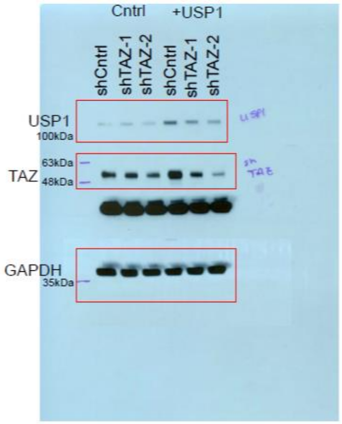

Figure 5E: shTAZ in USP1-OE MDA-MB-231

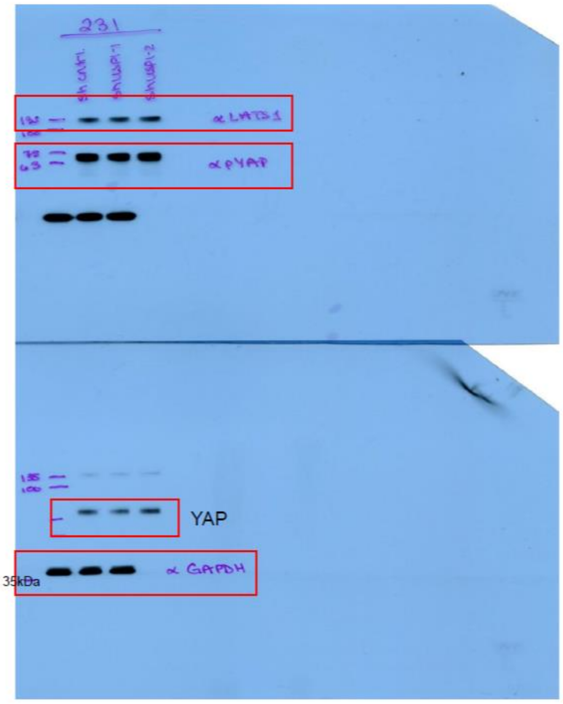

S1A: shUSP1 in MDA-MB-231 Hippo genes
